# Supplementary material for: MetaRibo-Seq measures translation in microbiomes
Source: Nat Commun. 2020 Jun 29;11:3268. doi: 10.1038/s41467-020-17081-z (PMC7324362; doi:10.1038/s41467-020-17081-z)
Supplement: Supplementary file 10 — Supplementary Data 7 [file 41467_2020_17081_MOESM10_ESM.zip › File2/Confidence_High_Taxonomy/201527_out.krona.html]

Javascript must be enabled to view this page.

members
magnitude
magnitudeUnassigned
count
unassigned
taxon
rank

201527\_out

6

2
superkingdom
2

1239
phylum
2

2
class
91061


SRS053356\_contig\_number\_23931SRS1041145\_contig\_number\_contig-100\_622.144593
1385
order
2

2759
1
superkingdom

kingdom
1
4751

451864
1
subkingdom

1
phylum
4890

147538
1
subphylum

1
class
147550

222543
1
subclass

order
1
5125

family
1
474942

genus
1
474995


SRS098571\_contig\_number\_43529
2004952
species
1

3

SRS013687\_contig\_number\_5705SRS023914\_contig\_number\_contig-100\_2940.146650SRS051031\_contig\_number\_contig-100\_3118.181022
